# Processing covert dependency: An eye-tracking study of scope interpretations of embedded *Wh*-questions in Mandarin

**DOI:** 10.1371/journal.pone.0285873

**Published:** 2023-05-18

**Authors:** Deran Kong, Yu-Yin Hsu

**Affiliations:** Department of Chinese and Bilingual Studies, The Hong Kong Polytechnic University, Hong Kong, Hong Kong; Universite de Geneve, SWITZERLAND

## Abstract

Non-local dependency in Mandarin *wh*-questions has been extensively researched in theoretical linguistics, but it remains an under-studied topic in the field of language processing. Unlike languages that require *wh*-movement to form *wh*-questions, Mandarin is a *wh*-in-situ language, and hence is generally assumed to require a covert dependency between a *wh*-phrase and its scope-bearing position. Mandarin therefore provides an ideal linguistic environment in which to study not only cognitive-processing mechanisms, but also how different types of non-local dependency, especially covert dependency, can be handled by readers. This paper investigates the processing of such covert non-local dependency in multiple embedded clauses, that is, multiple complementizer phrases (CPs). In *wh*-in-situ sentences with multiple CPs, the *wh*-phrases’ scope varies according to the types of verbs and their embedded clauses. Based on the subcategorization of clausal verbs, we designed four experimental conditions: double-embedded low scope, double-embedded high scope, double-embedded ambiguous scope, and long distance in pivotal construction. According to memory-based and distance-based language processing theories, the low-scope condition should be easier to process than the high-scope one, because the former has a shorter linear distance than the latter when forming dependencies; and pivotal construction should be easier to process than high-scope embedded clauses, because the former has a shorter structural distance. In cases where both low- and high-scope interpretations are possible, we aim to determine whether readers exhaust every potential interpretation during comprehension, or adopt a ‘good-enough’ approach to obtaining an interpretation via an easier and less costly process. To this end, we will adopt the eye-tracking technique that allows us to obtain fine-grained reading-time data, which can be used to compare processing across conditions. The results will contribute to understanding human readers’ mechanisms for processing covert dependency and resolving scope ambiguity in *wh*-in-situ languages.

## Introduction

A structural or interpretive association between two units in a sentence is referred to as *dependency*. When readers encounter a sentence, they need to incrementally identify, construct, and complete the structural-semantic dependencies among words in that sentence to obtain at least one acceptable interpretation. The parsing of non-local dependency, e.g., dependency between *wh*-phrases and the locus of their scope, is one of the most popular topics of research on sentence processing and language comprehension. As well as the mechanisms by which such dependencies can be constructed, researchers are especially interested in what may influence or interfere with parsing them during comprehension.

Mandarin is a *wh*-in-situ language, but its scope interpretations of *wh*-questions are parallel to those of languages employing *wh*-movements [1, 2]. The difference between these two language types is that while English, for example, overtly shows the interpretive association of *wh*-phrases, via the dislocation of such phrases to their interpretive position, Mandarin does so covertly. This difference is demonstrated by the English sentence in (1) and the Mandarin sentence in (2).

(1) The parents understand **which knowledge** the student has learned.
(2) 家长 清楚 ______ 学生们 学习了 **哪些 知识**.
  jiazhang qingchu ______ xueshengmen xuexile naxie zhishi
  parent understand ______ students learn-ASP which knowledge
  ‘The parents understand **which knowledge** the student has learned.’

Unlike (1), in which the *wh*-phrase ‘which knowledge’ moves to the beginning of the embedded clause, i.e., behind the verb ‘understand’, the same type of embedded *wh*-question in Mandarin allows the *wh*-phrase to be in its canonical, post-verbal position. To explain how the intended interpretation of a Mandarin sentence like (2) is obtained on a par with its English counterpart, it is generally assumed that there is a covert dependency between the empty, scope-interpretive position of a *wh*-phrase (marked by _____ as in (2)) and the intended *wh*-phrase located in the canonical position.

However, since such a *wh*-phrase remains in its post-verbal canonical position, its scope can be ambiguous, such as when more than one embedded clause (CP) is involved. As shown in (3a), the *wh*-phrase’s interpretive position can be in the higher clause (indicated by CP1), leading to an interpretation that ‘The teacher knew which knowledge the parents understood the students had learned.’ Alternatively, the interpretive position of the *wh*-phrase can be within the lower embedded clause (indicated by CP2), as in (3b). Under the latter interpretation, the teacher knew that the parents were clear about the students’ knowledge, but the teacher may or may not have known exactly which knowledge the parents knew the students had learned. In other words, not only can the scope interpretations of the *wh*-phrase in sentences like (3) be different, but also, the meaning of the entire sentence can be ambiguous, as demonstrated in (3a) and (3b). Because the empty interpretive positions in (3a) and (3b) are at different syntactic levels, dependency maintained across clauses (as in (3a)) is referred to as ‘high scope’, and dependency within the second clause (as in (3b)) is referred to as ‘low scope’.

(3) a. High scope
  老师 知道 [_CP1_ ______ 家长 *清楚* [_CP2_ 学生们 学习了
  laoshi zhidao ______ jiazhang *qingchu* xueshengmen xuexile
  teacher know ______ parent *understand* students learn-ASP
  **哪些 知识**.]]
**naxie zhishi**
  **which** knowledge
  ‘The teacher knew **which knowledge** the parents *understood* the students had learned.’
  b. Low scope
  老师 知道 [_CP1_ 家长 *清楚* [_CP2_ ______ 学生们 学习了
  laoshi zhidao jiazhang *qingchu* ______ xueshengmen xuexile
  teacher know parent *understand* _________ students learn-ASP
  **哪些 知识**.]]
**naxie zhishi**
**which knowledge**
  ‘The teacher knew the parents *understood* **which knowledge** the students had learned.’

In (3), the matrix verbs *知道 zhidao* (‘know’) and the embedded verb *清楚 qingchu* (‘understand’) both take interrogative clauses as their complements, resulting in scope ambiguity. However, some clausal verbs only take declarative clauses as their complements. When such verbs are used, some scope interpretations may therefore become unavailable, leading to an unambiguous interpretation. For example, the embedded verb *觉得 jude* (‘think’) in (4) cannot take an interrogative complement, and thus the low-scope interpretation of the *wh*-phrase in its CP2 is not possible; and its matrix verb *知道 zhidao* (‘know’) takes an interrogative complement, so the *wh*-phrase has to be interpreted in CP1.

(4) 老师 知道 [_CP1_ ______ 家长 *觉得* [_CP2_ 学生们 学习了
  laoshi zhidao ______ jiazhang *juede* xueshengmen xuexile
  teacher know ______ parent think students learn-ASP
  **哪些 知识**.]]
**naxie zhishi**
**which knowledge**
  ‘The teacher knew **which knowledge** the parents *thought* the students had learned.’

The embedded verb *好奇 haoqi* (‘wonder’) in (5), on the other hand, requires an interrogative clause to be its complement, and thus, the *wh*-phrase resolves its dependency within its CP2; and the matrix verb *知道 zhidao* (‘know’) therefore takes the whole double clause as its complement. As compared with (4), where the *wh*-phrase needs to be resolved in CP1, the *wh*-phrase’s interpretive position in (5) is lower, i.e., in CP2.

(5) 老师 知道 [_CP1_ 家长 *好奇* [_CP2_ ______ 学生们 学习了
  laoshi zhiadao jiazhang *haoqi* ______ xueshengmen xuexile
  teacher know parent *wonder* ______ students learn-ASP
  **哪些 知识**.]]
**naxie zhishi**
**which knowledge**
  ‘The teacher knew the parents *wondered* **which knowledge** the students had learned.’

The different interpretive positions of the embedded *wh*-phrase lead to varying processing loads when readers try to resolve dependency as part of sentence comprehension. Some memory-based and distance-based language-processing theories predict the relative processing difficulties of interpreting *wh*-phrases with high- or low-scope readings. For example, the Memory Retrieval account [3] holds that language processing is influenced by the time and effort it takes to retrieve a relevant element from the reader’s working memory. Because long-distance dependencies require such a relevant element to be stored in working memory, it is more difficult to retrieve than shorter-distance dependencies’ elements. In addition, the longer the dependency distance, the more interference there can be between the two dependent elements. As such, the Memory Retrieval account predicts that a *wh*-phrase with a high interpretive scope will require more memory load than one with a low scope; and therefore, that sentences like (4) will be more difficult to process than sentences like (5).

The Dependency Locality Theory [4, 5] suggests a similar distance effect: the greater the distance between the two dependent elements is, the higher the processing cost will be. Nonetheless, it must be borne in mind that the distance between two dependent elements can be measured either linearly (as the number of words that separate them), or structurally (as the number of hierarchical-phrasal boundaries between them). For instance, the linear distance between the *wh*-phrase and its interpretive scope in (6) is the same as in (4). However, the embedded verb *帮助 bangzhu* (‘help’) in (6) is part of a pivotal construction resulting in the stacking of two verb phrases in one clause [6–8], rather than two embedded clauses, as in (4). Therefore, despite the linear distance being the same, the structural distance in a pivotal construction like that of (6) should be considered shorter or smaller than such distance in a sentence with a doubly embedded clause, e.g., (4). By using items like examples (4) and (6) in experiments, we can further explore questions around linear vs. structural distance that are relevant to the validity of memory-retrieval theories.

(6) 老师 知道 [_CP1_ ______ 家长 *帮助* 学生们 学习了
  laoshi zhidao jiazhang *bangzhu* xueshengmen xuexile
  teacher know ______ parent *help* students learn-ASP
  **哪些 知识**.]]
**naxie zhishi**
**which knowledge**
  ‘The teacher knew **which knowledge** the parents *helped* the students to learn.’

In summary, a comparison between examples like (4) and (5) goes some way to addressing the different linear distances in sentence processing, while a comparison between (4) and (6) illustrates the issue of linear vs. structural distances. All three of these sentences, however, are supposed to have *wh*-phrases with unambiguous scopes.

Yet, according to the Minimal Chain Principle of processing *wh*-questions [9], a comprehender will avoid positing a gap when a filler has yet to be encountered; but when a dependency relation is required, the process of forming such a dependency should be as short as possible. In line with this principle, one can expect a parser to start to predict and hold a gap after reading verbs like ‘wonder’ in (5) that require a *wh*-phrase as the filler, whereas no such gap-filler expectation will occur in other conditions because such sentences can be ambiguous in either a long-distance matrix *wh*-reading (because the need for a filler only shows up in the very last words of a sentence) or a non-interrogative reading (because no gap-filler dependency is needed). Therefore, by the Minimal Chain Principle, gap-activation as in sentences like (5) may require more memory effort than processing sentences like (4) and (6) in which a no-dependency reading is allowed before the *wh*-phrase is reached.

Another critical point in processing sentences with a covert dependency of embedded *wh*-questions is that some require the resolution of ambiguous scope readings. As mentioned earlier, sentences like (3) allow both high and low interpretive positions of the *wh*-phrase. If the two ambiguous scopes are processed in parallel [10–12], readers will examine both the high- and low-scope interpretations before their own interpretation is finalized. In such cases, the processing load would be large, because the reader needs to consider a long-distance dependency (high scope). However, it is also possible that once readers using heuristic parsing [13] feel they can adopt a ‘good-enough’ interpretation [14–17], they may not exhaust all potential interpretations. More specifically, once readers find that a low-scope interpretation of a *wh*-phrase is acceptable, they will not tend to try the high-scope interpretation, as doing so would require more memory effort. In such cases, the processing load of a heuristic-parsing approach can reasonably be expected to be less than that of the embedded *wh*-phrases requiring high scope, insofar as only a shorter-distance dependency needs to be resolved. To clarify that point, this paper investigates which strategy (parallel processing vs. ‘good-enough’ heuristic parsing) readers tend to adopt when trying to comprehend embedded *wh*-questions with ambiguous scopes.

To the best of our knowledge, only two studies have previously focused on both the processing and the structural issues related to covert Mandarin *wh*-in-situ dependency. Xiang et al. (2015) [18] designed four experimental conditions. Of these, the high-scope one (as (4)) featured multiple CPs, while the other three differed in terms of their linear distances. The authors found an interference effect in the processing of *wh*-in-situ sentences, supporting the distance-based accounts [3–5]. In the same study, Xiang et al. also reported that their condition with multiple CPs was the most difficult one when it came to processing non-local dependency, due to interference from the verbs of the embedded clauses. However, that paper considered only one type of multiple CPs. In a subsequent study, Xiang et al. (2023) [19] looked at two types of *wh*-in-situ sentences with multiple CPs to investigate the resolution of *wh*-scope ambiguity. That work established that, while one of these types only allowed a high-scope interpretation, the other allowed both high- and low-scope ones. In its experiments, the participants were first provided with a context to disambiguate scope, and then were asked to judge whether the critical (i.e., multiple-CP) sentences were true or false according to a further elaborated context. The results showed that, although there was a bias towards low scope in terms of reaction time, the participants’ interpretations did not favor the low scope. In the same paper, Xiang et al. indirectly explained this discrepancy through pragmatic reasoning, including that the design could have been confounded by some scope-biased interpretations that were introduced by the elaborated contexts. More specifically, such contexts required the participants to arrive at either a low- or high-scope interpretation, and thus determine the ‘right answer’ in a true-or-false judgment task. However, it remains unclear whether such scope information arose from the critical sentences themselves, or was a re-interpretation after reading contextual materials.

Although two studies have examined how native Mandarin speakers process and comprehend covert dependencies in embedded *wh*-questions, the differences between low- and high-scope processing remain in need of closer examination. In situations where both high- and low-scope interpretations are possible, it is still unclear whether readers tend to exhaust both possibilities in parallel parsing before arriving at what they consider to be the optimal interpretation [10–12], or use the ‘good-enough’ heuristic approach to reduce memory demand [13–17]. Given that biased contexts might have influenced the results of prior research, we propose a simpler paradigm—in which the basic context does not include cues for relevant scope interpretations—for studying how scope ambiguities are resolved.

As we have seen, previous studies have discussed how distance modulates memory burdens; but none have made a direct comparison between embedded *wh*-questions that have identical numbers of words, but different interpretive positions of the *wh*-phrases. Moreover, the role of structural distance deserves more attention. The present paper therefore investigates the processing of *wh*-questions in embedded pivotal constructions, with the aim of revealing the role of linear as well as structural distances during language comprehension. Its results can also be expected to provide new insights into how readers understand the syntactic structure of pivotal constructions containing covert dependencies. By comparing the processing of embedded *wh*-questions with high vs. low scope, and *wh*-questions in double-embedded clauses vs. embedded pivotal constructions, our study will provide important empirical evidence about the processing issues summarized above.

### The present study

Using Mandarin as an example of a *wh*-in-situ language that requires the processing of covert dependency and the resolution of scope ambiguity, this study seeks answers to the following three research questions:

1. Is processing low-scope embedded *wh*-questions easier than high-scope ones, as predicted by memory- and distance-based language-processing theories?
2. Given the same linear distances, is processing long-distance covert dependencies easier in embedded pivotal constructions than in doubly embedded clauses?
3. Do readers process covert dependencies by examining all possible readings, or by adopting a ‘good-enough’ heuristic strategy?

To answer these questions, we designed four experimental conditions. They were: a) double-embedded low scope; b) double-embedded high scope; c) double-embedded ambiguous scope; and d) long distance in pivotal construction.

According to the memory-based and distance-based theories reviewed above, the low-scope condition should make for easier processing than the high-scope one. A comparison between condition a) and condition b) will therefore provide the answer to our first research question. By adding a structurally simpler condition, d), we can answer the second research question by comparing it against condition a). As for the third research question, given that both low- and high-scope interpretations are possible in condition c), we aim to determine whether readers adopt the ‘good-enough’ strategy to arrive at an understanding more straightforwardly—much as in the low-scope condition, a)—or exhaust every potential interpretation. We will use the eye-tracking technique to obtain fine-grained reading-time and regression-pattern data to compare the processing of different conditions. The results will contribute to a more comprehensive understanding of the processing of covert dependency and the resolution of scope ambiguity in *wh*-in-situ languages.

## Materials and methods

### Participants

We used a power-analysis website [20] to estimate the minimal number of participants needed for a statistical power of 80%. The dataset we used for this power analysis is from our unpublished eye-tracking project on the processing of Mandarin relative clauses, with similar measures and designs to the present one (one fixed and two random effects). The values used in the power analysis were as follows: Effect size d: 0.17; Residual VPC: 0.79; Participant intercept VPC: 0.14; Stimulus intercept VPC: 0.04; Participant-by-Stimulus VPC: 0.01; Participant slop VPC: 0.02; Stimulus slop VPC: 0.00. The solution suggested a minimum requirement for 88 participants.

Thus, based on the results of a language-background questionnaire distributed online, 88 right-handed, healthy, normally developed native speakers of Mandarin from Mainland China who are attending college in Hong Kong will be invited to participate in this study in the speech lab of the Hong Kong Polytechnic University. Informed consent will be obtained from the participants before the start of data collection, and after completing the experiment, they will be compensated with HK$60.00 (about US$7.7).

### Materials

The four conditions for the critical sentences are shown in (7). A total of 16 sentence sets were created in simplified Chinese characters (see S1 File). The only difference among the conditions in each such set was the second verb, i.e., the first embedded verb. Because we have four conditions, a fully counterbalanced design would consist of four different lists with 16 sentences per list, and in each list, items from the same set would only appear once. However, such a design could only provide four items per participant per condition, which would lead to extremely low statistical power [21]. To solve this problem, we decided to let each participant finish two lists, divided by a break, and all the lists will be presented at the first half of the experiment or at the second half of the experiment the same number of times. It is reasonable to question this ‘two lists per participant’ design as potentially leading to repetition effects, as similar words will be encountered in the second list. We would argue, however, that even though there are repetitions, any repetition effects should be the same for all conditions, and thus will be effectively counterbalanced when we make the comparisons between conditions that are the main focus of the proposed experiment. There will also be 48 filler sentences to mask the true purpose of the experiment. Thus, each participant will read a total of 80 sentences.

(7) 在这学期的期末家长会上,
  zai zhe xueqi de qimo jiazhanghui shang,
  ’At this semester’s term-end teacher-parent meeting,’

a double-embedded low scope
  老师 知道 [_CP1_ 家长们 *好奇* [_CP2_ 学生们 学习了 **哪些 知识**.]]
  laoshi zhidao jiazhangmen *haoqi* xueshengmen xuexile **naxie zhishi**
  teacher know parents *wonder* students learn-ASP **which knowledge**
  ‘The teacher knew the parents *wondered* **which knowledge** the students had learned.’
b double-embedded high scope
  老师 知道 [_CP1_ 家长们 *觉得* [_CP2_ 学生们 学习了 **哪些 知识**.]]
  laoshi zhidao jiazhangmen *juede* xueshengmen xuexile **naxie zhishi**
  teacher know parents *think* students learn-ASP **which knowledge**
  ‘The teacher knew **which knowledge** the parents *thought* the students had learned.’
c double-embedded ambiguous scope
  老师 知道 [_CP1_ 家长们 *清楚* [_CP2_ 学生们 学习了 **哪些 知识**.]]
  laoshi zhidao jiazhangmen *qingchu* xueshengmen xuexile **naxie zhishi**
  teacher know parents *understand* students learn-ASP **which knowledge**
  ‘The teacher knew the parents understood **which knowledge** the students had learned.’ (low scope)
  ‘The teacher knew **which knowledge** the parents understood the students had learned.’ (high scope)
d long distance in pivotal construction
  老师 知道 [_CP1_ 家长们 *帮助* 学生们 学习了 **哪些 知识**.]
  laoshi zhidao jiazhangmen *bangzhu* xueshengmen xuexile **naxie zhishi**
  teacher know parents *help* students learn-ASP **which knowledge**
  ‘The teacher knew **which knowledge** the parents had *helped* the students learn.’

The second verb/first embedded verb in each sentence (i.e., the italicized verb in CP1) takes one of the four types of complements. In condition a), the *好奇 haoqi* (‘wonder’) type of verb requires an interrogative complement, so the *wh*-phrase is resolved as its complement within CP2, forming a low-scope dependency. The *觉得 juede* (‘think’) type of verb in condition b), in contrast, does not allow an interrogative complement, blocking the low-scope interpretation. As a result, the *wh*-phrases can only build a dependent relationship with the gap after the matrix verb *知道 zhidao* (‘know’), forming a high-scope dependency. In condition c), the *清楚 qingchu* (‘understand’) type of verb *allows*, but does not definitely *require*, an interrogative complement; therefore, both high- and low-scope interpretations are possible. In condition d), the *帮助 bangzhu* (‘help’) type of verb does not form an additional embedded CP right after the verb. Thus, although the linear distance between the filler and gap in condition d) is the same as in condition b), in the high-scope condition, the structural distance that the *wh*-phrase needs to cross is different. Specifically, in condition d), the *wh*-phrase only needs to form the dependency within CP1, while in condition b), it must cross CP2 and fill the gap after the matrix verb. The manipulation of scope readings enables us to investigate readers’ processing of non-local dependencies with different linear distances (i.e., condition a) vs. condition b)); ambiguous scopes (condition c)); and the same linear distances, but different structural distances (condition b) vs. condition d)).

Next, we conducted a norming test to confirm whether the different types of verbs we had selected actually belonged to the categories we claim they do [22]. We assumed that, in condition a), each manipulated (i.e., second/italic) verb required an interrogative as its complement, and rejected a declarative one. To the extent that this assumption was correct, people should have accepted sentences like (8a) and rejected sentences like (8b). The manipulated verbs in conditions b) and d), in contrast, cannot take interrogatives. Therefore, sentences with *wh*-phrases as their complements, like (9a) and (11a), should be rejected; but those with definite nouns as their complements, e.g., (9b) and (11b), should be seen as legitimate. The manipulated verbs in condition c), meanwhile, should be able to take either interrogative or declarative complements, given that this condition was designed to allow ambiguous scope interpretation. Lastly, the matrix verb takes a declarative CP as its complement in condition a), and an interrogative one in condition b). Therefore, the matrix verb should also allow as its complement either an interrogative or a declarative one, of the same categories as the manipulated verbs in condition c).

(8) a. 家长们 *好奇* 学生们 学习了 **哪些 知识**.
  jiazhangmen *haoqi* xueshengmen xuexile naxie zhishi
  parents *wonder* students learn-ASP **which knowledge**
  ‘The parents *wondered* **which knowledge** the students had learned.’
  b. *家长们 *好奇* 学生们 学习了 **那些 知识**.
  jiazhangmen *haoqi* xueshengmen xuexile **naxie zhishi**
  parents *wonder* students learn-ASP **those knowledge**
  ‘*The parents *wondered* the students had learned **that knowledge**.’
(9) a. *家长们 *觉得* 学生们 学习了 **哪些 知识**.
  jiazhangmen *juede* xueshengmen xuexile **naxie zhishi**
  parents *think* students learn-ASP **which knowledge**
  ‘*The parents *thought* **which knowledge** the students had learned.’
  b. 家长们 *觉得* 学生们 学习了 **那些 知识**.
  jiazhangmen *juede* xueshengmen xuexile **naxie zhishi**
  parents *think* students learn-ASP **those knowledge**
  ‘The parents *thought* the students had learned **that knowledge**.’
(10) a. 家长们 *清楚* 学生们 学习了 **哪些 知识**.
  jiazhangmen *qingchu* xueshengmen xuexile **naxie zhishi**
  parents *understand* students learn-ASP **which knowledge**
  ‘The parents *understand* **which knowledge** the students had learned.’
  b. 家长们 *清楚* 学生们 学习了 **那些 知识**.
  jiazhangmen *qingchu* xueshengmen xuexile **naxie zhishi**
  parents *understand* students learn-ASP **those knowledge**
  ‘The parents *understand* the students learned **that knowledge**.’
(11) a. *家长们 *帮助* 学生们 学习了 **哪些 知识**.
  jiazhangmen *bangzhu* xueshengmen xuexile **naxie zhishi**
  parents *help* students learn-ASP **which knowledge**
  ‘*The parents *helped* the students to learn **which knowledge**.’
  b. 家长们 *帮助* 学生们 学习了 **那些 知识**.
  jiazhangmen *bangzhu* xueshengmen xuexile **naxie zhishi**
  parents *help* students learn-ASP **those knowledge**
  ‘The parents *helped* the students to learn **that knowledge**.’

To test whether our verb categorization was consistent with native speakers’ judgments, 42 native Mandarin speakers provided ratings of these sentences in an acceptability judgment task (AJT), on a five-point Likert scale ranging from 1 = ‘totally unacceptable’ to 5 = ‘totally acceptable’. Verbs whose ratings were not in line with our assumptions were deleted or re-tested. In the end, we were left with 11 ‘wonder’ verbs as in (8), 11 ‘think’ verbs as in (9), 24 ‘understand’ verbs as in (10), and 12 ‘help’ verbs as in (11). The mean ratings for each sentence can be found in our S1 File. We found that in some sentences, if the verbs are modified by an aspect marker, sentence naturalness improves, but in others, bare verbs without any aspect markers are more suitable. Investigating the reason(s) for this phenomenon is not the focus of this study. Yet, we want our experimental sentences to be as natural as possible. Therefore, in the norming test, we asked the participants to rate the acceptability of the same sentence twice, one with and the other without an aspect marker attached to the matrix verb. The version with a higher rating was then used to develop the experimental sentences.

As shown in (7), before each critical sentence, the participants were shown a concise preceding context, for example, *在这学期的期末家长会上 zai zhe xueqi de qimo jiazhanghui shang* (‘At this semester’s term-end teacher-parent meeting’). We decided to provide such context for the following two reasons. First, our preliminary acceptability rating results showed that the ratings of single *wh*-in-situ sentences with multiple CPs tended to be low when no context was provided. This was understandable, as these sentences were long and required readers to retrieve non-local cues. Difficulties in processing can result in low acceptability [23], and concise context information helps participants make sense of critical sentences, facilitating their parsing and interpretation. Second, our intent was to design contexts that were not biased either toward a low-scope or a high-scope interpretation, to avoid potential contextual influences of the type observed in [23]. Providing unbiased contexts will enable us to explore patterns of non-local dependency processing when there is more than one potential dependent element.

It is worth mentioning that since *wh*-words remain in their canonical position in both matrix and embedded questions, punctuation is essential to correct interpretation of Mandarin questions. All our experimental sentences are embedded *wh*-questions, so all end with a period, as in (12). However, the same sentence could also be interpreted as a matrix question if a question mark is placed at the end, as shown in (13). To avoid readers misinterpreting the embedded questions as matrix questions, instructions will be given to stress that special attention to punctuation is required. And, after the experiment, the participants will be asked whether they have encountered any sentences ending with a question mark. Because all the experimental and filler sentences will end with a period, participants who answer ‘Yes’ may have interpreted the doubly embedded questions as matrix questions, and their data will be excluded from the analysis.

Sentences in the norming test face the same problem of readers mistaking them for matrix questions due to inattention to punctuation. The above-mentioned procedures were therefore also adopted in the norming test.

(12) 老师 知道 家长们 好奇 学生们 学习了 **哪些 知识**.
  laoshi zhidao jiazhangmen haoqi xueshengmen xuexile **naxie zhishi**
  teacher know parents wonder students learn-ASP **which knowledge**
  ‘The teacher knew the parents wondered **which knowledge** the students had learned.’
(13) 老师 知道 家长们 好奇 学生们 学习了 **哪些 知识?**
  laoshi zhidao jiazhangmen haoqi xueshengmen xuexile **naxie zhishi**
  teacher know parents wonder students learn-ASP **which knowledge**
  ‘**Which knowledge** did the teacher know the parents wondered the students had learned?’

### Procedure

Eye-tracking recording with an eyetracker (SR Research, Eyelink 1000 Plus) will be used in this study to obtain time-sensitive observations of participants’ processing of *wh*-in-situ sentences with multiple CPs. The materials will be presented on a 1024 x 768 pixel LCD display monitor, and text will be displayed in black against a light-grey background. The system features high spatial resolution (<0.5◦) and a sampling rate of 1000 Hz. The experiment will be set up using SR Research’s Experiment Builder, and the eye-tracking measurements will be analysed and extracted using SR Research’s Data Viewer.

Each participant will sit in a comfortable chair with its height individually adjusted. Before starting, they will be instructed to minimize their head movements. During the experiment, each participant will lean onto an individually adjusted chin-and-forehead rest, positioned 80cm away from the screen, to facilitate relaxation and further minimize head movements. A nine-point calibration procedure will be performed at the start of the experiment. The participant will trigger the display of each sentence by fixating on a dot at the upper left edge of the monitor.

The participant will read the sentences one by one at their own speed while their eye movements are recorded. After each sentence, the participant will be asked a yes/no comprehension question about the sentence they just read. As well as to provide data on comprehension performance, the comprehension questions are designed to confirm that the participants remained focused when parsing the trials shown on the screen.

The whole experiment will include 80 trials (i.e., 32 critical items and 48 fillers). All sentences will be displayed in simplified Chinese characters on two lines: the first being the context, and the second, the critical sentence (at the center of the screen). The font will be Songti SC with a size of 40 points. To avoid fatigue, there will be a pause after 40 trials, during which the participants can take a break and resume the experiment when they are ready or after 5 minutes, whichever is less. Thus, the total length of a test session, including preparation and eye-tracking calibration, will be approximately 45 minutes per participant.

### Data-exclusion criteria

We decided in advance that participants and data points would be excluded under certain conditions. First, all data from a given participant will be excluded if they have an accuracy rate lower than 80% for the comprehension questions, as this will indicate that they were probably not focused enough during the experiment. Second, data cleaning will be performed using the four-stage fixation cleaning procedure in Data Viewer. The thresholds are: 80ms, 0.5° (Stage 1); 40ms, 1.25° (Stage 2); 140ms (Stage 3); <140ms, >800ms (Stage 4). Third, we will perform a priori screening in combination with model criticism to identify outliners, following the recommendations [24]. This a priori screening will consist of examining by-subject log-transformed Q-Q plots of the reaction times (RTs) for each individual. Data points that deviate from the normal distribution curve too much will be treated as outliers and deleted. Next, a preliminary model will be built using the trimmed data, and its residuals inspected to establish whether it is stressed. If it fails to fit longer latencies well, data points with absolute standardized residuals exceeding 2.5 standard deviations will be moved. A new model will be built using the data after this process of model criticism. If the preliminary model fits all the data well, no further data cleaning will occur.

### Statistical-analysis plan

Four regions—the matrix verb, the first embedded verb, the *wh*-word, and the word following the *wh*-phrase (which is also the last word of the sentence)—will form our interest areas (IAs). The matrix verb (IA1) is the location where the covert dependency is assumed to be realized when scope interpretation is high. The first embedded verb (IA2) is the critical location when readers make a low-scope interpretation; and the *wh*-phrase (IA3 and IA4) is the filler position where the parser encounters the *wh*-phrase for the first time.

We will choose four durational metrics and two regression-count ones, which are described in turn below. **First-fixation duration (FFD)**: the duration of the first fixation event located in this IA. **First-pass time (FPT)**: the sum of the durations across all fixations in this IA in the first run, from when this IA is first entered until it is exited. **Regression-path duration (RPD)** is the sum of the durations across all fixations from when the current IA is first entered until it is exited to the right, including the fixations located outside of it. **Total reading time (TRT)** is the sum of all the durations across all fixations located in the IA. **The probability of regression-in (R-IN)** and **the probablility of regression-out (R-OUT)**. Regression-in indicates the current IA was entered from the right of this IA, i.e., from later in the sentence; and Regression-out indicates the current IA was exited to the left, i.e., into the earlier part of the sentence. We will then measure the probability of existing or accessing a region or not of R-IN and R-OUT.

We will use different mixes of metrics that are relevant to our predictions. In the case of IA1—the gap position for high-scope interpretations—the gap is not activated until a filler (the *wh*-phrase) is encountered. Thus, for IA1, we will only adopt TRT and R-IN, as the other metrics are more applicable after later parts of the sentence have been read.

For the gap position for low-scope interpretations, IA2, FFD and FPT will be adopted. According to the Minimal Chain Principle (De Vincenzi, 1991), in this region (the first embedded verb), only condition b) activates the gap, while other conditions wait until the filler (the *wh*-phrase) is encountered. Gap activation may lead to longer fixations for condition b) at this stage. However, after the filler is encountered, this early gap activation may facilitate the integration of the filler-gap dependency for condition b). Therefore, the effect may be neutralized on TTR. As a result, we predict the effects of early gap activation will be more pronounced in early-stage eye-tracking measures (FFD and FPT).

For IA3 and IA4, where the *wh*-phrase is encountered, we plan to use RPD, TRT, and R-OUT to measure the processing loads of resolving long-distance gap-filler dependency. When readers encounter a *wh*-phrase at a sentence’s end, they need to find an interpretive position for it. The search for such a position could be reflected by RPD and R-OUT, and the integration reflected by TRT.

As we will be using more than one metric for each IA, the multiple comparisons problem arises, and alpha levels need to be corrected [25]. As the predicted effects for each IA are based on theories reflecting different processing mechanisms as the sentence unfolds, the corrections for alpha levels are calculated for each IA rather than globally. For IA1 (TRT and R-IN), the alpha level will be 0.05/2 = 0.025. For IA2 (FFD and FPT), the alpha level will also be 0.025. For IA3 and IA4, three measures (RPD, TRT, and R-OUT) will be used, so the alpha level will be .05/3 = .017. The above-cited study also mentions that eye-tracking metrics are rarely uncorrelated. Therefore, we will include a correlation matrix of the metrics we use.

Mixed-effects models using the lme4() package in R will be developed to analyze the eye-movement data. The above-mentioned metrics will be the response variables, and the three predictors will be condition (fixed effect) and participant and item (random effects). For IA2, the number of Chinese characters per sentence differs across the conditions for some items. The response time measures will therefore be length-corrected by using the residuals of the model with WORD LENGTH as a function of the original response time [26].

The maximal model will be the one with both random intercepts (item and participant) and random slopes (by condition), as we allow different participants and items to have different RTs, and because we allow the change between conditions to be different for each participant and item. That is, the maximal model can be expressed as RT ~ condition + (condition|item) + (condition|participant). The other five possible models with simpler random effects are RT ~ condition + (1|item) + (condition|participant); RT ~ condition + (condition|item) + (1|participant); RT ~ condition + (1|item) + (1|participant); RT ~ condition + (1|item); RT ~ condition + (1|participant). We will compare all the models that successfully converge and choose the one with the lowest Akaike Information Criterion (AIC) [27].

### Predictions

RQ1 addresses the linear-distance effect on the processing of non-local dependencies. According to memory-based retrieval accounts, long distances and interference between dependent elements should trigger difficulties in processing such dependencies. In condition b), the linear distance between the *wh*-phrases and the empty position at its interpretive scope is longer than in condition a). Also, the embedded verb in condition b) appears between the two dependent elements and is thus more likely to cause interference. Thus, we expect longer reading times and more regressions for condition b) than for condition a) at IA1 (the gap position for condition b)), and at IA3 and IA4 (the filler positions).

However, the Minimal Chain Principle predicts that in sentences like those that make up condition a), an early gap-filler activation (at IA2) will make processing more difficult, because the reader has to hold the dependency and search for the filler, resulting in longer processing times than in conditions that do not require a dependency to be imposed until the filler is encountered. Thus, the early-stage processing time at IA2 (the gap position for condition a)) is expected to be longer for condition a) than for the other conditions.

RQ2 involves the effect of syntactic structure on the processing of long-distance dependency. Although condition d) and condition b) share the same linear distance between the *wh*-phrase and its interpretive position, the *wh*-phrase in condition d) is in a pivotal construction, which is believed to have a simpler structure, and thus to cause less interference, than double-embedded clauses. Therefore, we predict that the processing of condition b) will be harder than that of condition d), resulting in shorter reading times and fewer regressions for the latter than for the former at IA1, IA3, and IA4. IA2 is irrelevant to research question 2, because neither condition b) nor condition d) should include gap-filler activation at that position.

As for RQ3, there are two potential processing patterns for condition c). If readers apply the ‘good-enough’ approach to interpreting the sentences, they will use simple heuristics to arrive at an interpretation more straightforwardly, which we predict will be a low-scope interpretation requiring fewer memory resources. Thus, if a ‘good-enough’ approach is used, the reading times and regression patterns of condition c) should be similar to those of condition a) at IA1, IA3 and IA4. On the other hand, if readers process both high and low scope in parallel before arriving at their final interpretations, they will require more time, not only to keep both interpretations active, but also to then decide between two valid interpretations. Thus, in this circumstance, we predict that the reading time for condition c) will be the longest at IA1, IA3, and IA4.

**Fig 1:**
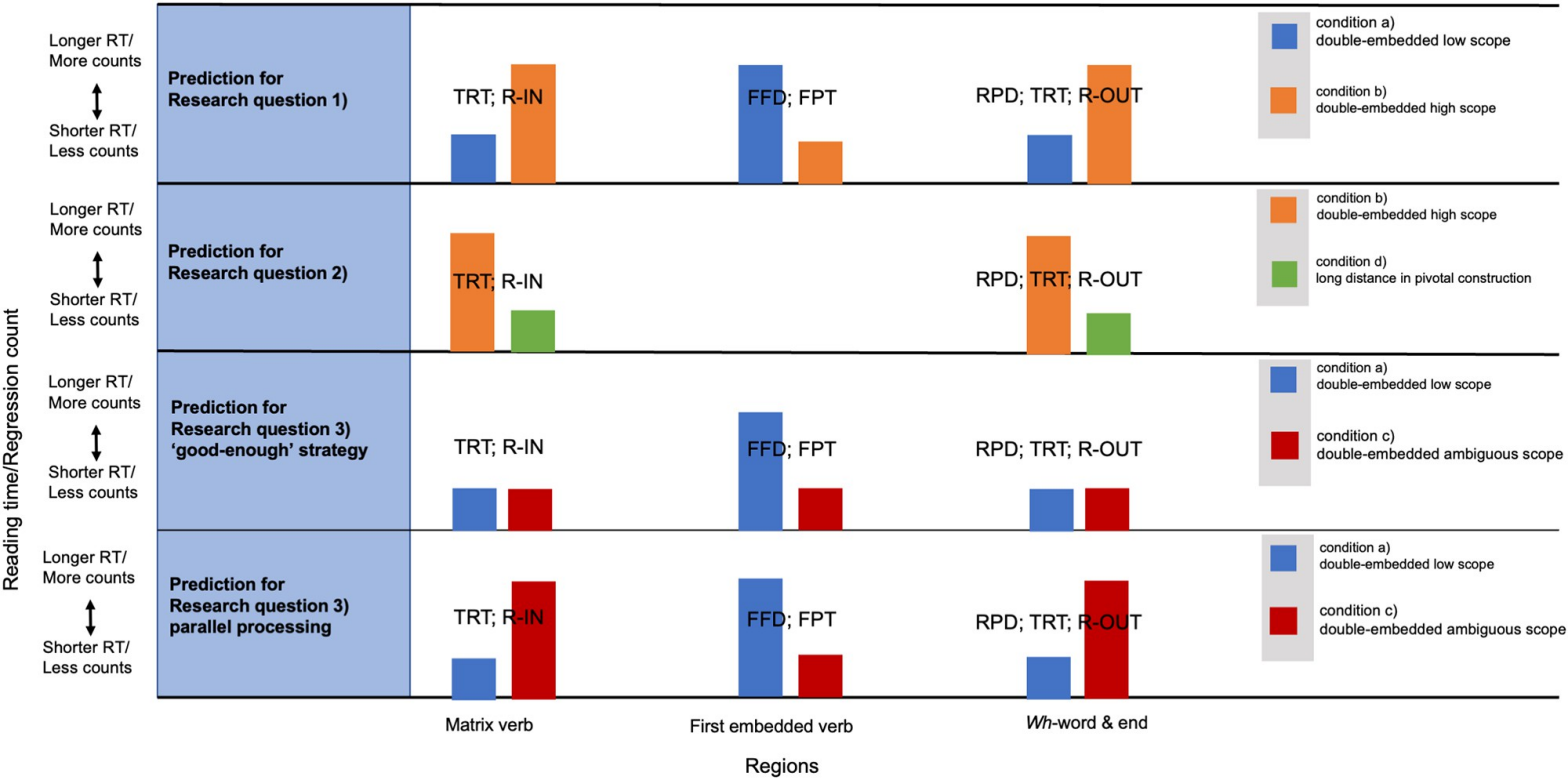
Predicted effects in different regions for each research question.

The predictions for each research question are visualized in Fig 1, in which the above-mentioned abbreviations for the eye-tracking metrics we intend to adopt are provided for each IA.

## Results

…

### Timeline

A review of the ethics of this human-subjects experiment was conducted and signed of by the host university (PolyU; approval: HSEARS20210315014). With the assumption that this Registered Report Protocol is accepted, we plan to collect data during spring 2023, complete the data analysis by fall 2023, and we will submit the paper by winter 2023.

## Supporting information

S1 File Experimental materials, norming results. (ZIP)
