# Supplementary material for: Processing covert dependency: An eye-tracking study of scope interpretations of embedded Wh-questions in Mandarin
Source: PLoS One. 2023 May 18;18(5):e0285873. doi: 10.1371/journal.pone.0285873 (PMC10194922; doi:10.1371/journal.pone.0285873)
Supplement: S1 File — (ZIP) [file pone.0285873.s001.zip › supporting infomration/Experimental Materials.pdf]

Experimental Materials

1.	Context.	在环保组织的新闻发布会后，<br>After the press conference organized by the environmental group,
	a.	记者报道了研究员*想知道*环保队采集了**哪些树种**。<br>the reporter reported the researchers *wanted to know* **which tree species** the environmental protection team had collected.
	b.	记者报道了研究员*怀疑*环保队采集了**哪些树种**。<br>the reporter reported **which tree species** the researchers *suspected* the environmental protection team had collected.
	c.	记者报道了研究员*宣布了*环保队采集了**哪些树种**。<br>the reporter reported the researchers *announced* **which tree species** the environmental protection team had collected. (low)<br>the reporter reported **which tree species** the researchers *announced* the environmental protection team had collected. (high)
	d.	记者报道了研究员*辅助*环保队采集了**哪些树种**。<br>the reporter reported **which tree species** the researchers had *helped* the environmental protection team to collect.
	Question.	刚才提到的是一条八卦新闻。（错）<br>What has just been mentioned is a piece of gossip. (False)

2.	Context	在文化局对各单位的审查中，<br>In the Cultural Affairs Bureau’s reviews of different departments,
	a.	馆长发现了市领导*弄不清*博物馆收藏了**哪些古董**。<br>the curator discovered the municipal leaders *were confused about* **which antiques** the museum had collected.
	b.	馆长发现了市领导*以为*博物馆收藏了**哪些古董**。<br>the curator discovered **which antiques** the municipal leaders *believed* the museum had collected.
	c.	馆长发现了市领导*隐瞒了*博物馆收藏了**哪些古董**。<br>the curator discovered the municipal leaders *concealed* **which antiques** the museum had collected. (low)<br>the curator discovered **which antiques** the municipal leaders *concealed* the museum had collected. (high)
	d.	馆长发现了市领导*资助*博物馆收藏了**哪些古董**。<br>the curator discovered **which antiques** the municipal leaders had *subsidized* the museum to collect.
	Question	文化局对博物馆进行审查。（对）<br>The Cultural Affairs Bureau reviews the museum. (True)

3.	Context	在反腐新闻发布会上，<br>In a press conference about anti-corruption,
	a.	市长总结了群众们*想了解*市政府禁止了**哪些行为**。<br>the mayor summarized the people *wanted to know* **which behaviors** the city government had prohibited.
	b.	市长总结了群众们*相信*市政府禁止了**哪些行为**。<br>the mayor summarized **which behaviors** the people *believed* the city government had prohibited.
	c.	市长总结了群众们*听到*市政府禁止了**哪些行为**。<br>the mayor summarized the people *heard* **which behaviors** the city government had prohibited. (low)

		the mayor summarized **which behaviors** the people *heard* the city government had prohibited. (high)
	d.	市长总结了群众们*说服*市政府禁止了**哪些行为**。<br>the mayor summarized **which behaviors** the people had *persuaded* the city government to prohibit.
	Question	市长出席了这场新闻发布会。（对）<br>The mayor attended this press conference. (True)
4.	Context	在这学期的期末家长会上，<br>At this semester's term-end teacher-parent meeting,
	a.	老师知道家长们*好奇*学生们学习了**哪些知识**。<br>the teacher knew the parents *wonder* **which knowledge** the students had learned.
	b.	老师知道家长们*觉得*学生们学习了**哪些知识**。<br>the teacher knew **which knowledge** the parents *thought* the students had learned.
	c.	老师知道家长们*清楚*学生们学习了**哪些知识**。<br>the teacher knew the parents *understood* **which knowledge** the students had learned. (low)<br>the teacher knew **which knowledge** the parents *understood* the students had learned. (high)
	d.	老师知道家长们*帮助*学生们学习了**哪些知识**。<br>the teacher knew **which knowledge** the parents had *helped* the students to learn.
	Question	家长觉得老师们体罚了学生。（错）<br>The parents felt that the teachers were physically punishing the students. (False)
5.	Context	传言公司数据库丢失了信息，<br>There is a rumour that the company database has lost information.
	a.	秘书猜出了总经理*在调查*程序员删除了**哪些资料**。<br>The secretary guessed (out) the general manager *was investigating* **which data** the programmers had deleted.
	b.	秘书猜出了总经理*认为*程序员删除了**哪些资料**。<br>The secretary guessed (out) **which data** the general manager *believed* the programmers had deleted.
	c.	秘书猜出了总经理*知道*程序员删除了**哪些资料**。<br>The secretary guessed out the general manager *knew* **which data** the programmers had deleted. (low)<br>The secretary guessed out **which data** the general manager *knew* the programmers had deleted. (high)
	d.	秘书猜出了总经理*串通*程序员删除了**哪些资料**。<br>The secretary guessed (out) **which data** the general manager had *colluded with* the programmers to delete.
	Question	传言公司被偷走了大量现金。（错）<br>The rumour is that the company has lost a lot of cash. (False)
6.	Context	在最近一次的学术会议上，<br>At a recent academic conference,
	a.	教授报告了古人类*在探索*外星人建造了**哪些城市**。<br>the professor reported the ancient humans *were exploring* **which cities** the aliens had built.
	b.	教授报告了古人类*坚信*外星人建造了**哪些城市**。

		the professor reported **which cities** the ancient humans *believed* the aliens had built.
	c.	教授报告了古人类*明白*外星人建造了**哪些城市**。<br>the professor reported the ancient humans *knew* **which cities** the aliens had built. (low)<br>the professor reported **which cities** the ancient humans *knew* the aliens had built. (high)
	d.	教授报告了古人类*配合*外星人建造了**哪些城市**。<br>the professor reported **which cities** the ancient humans had *cooperated with* the aliens to build.
	Question	会议探讨外星人建城市的话题。(对)<br>This conference explored the topic of aliens building cities. (False)

7.	Context	娱乐小报称近期将爆料八卦，<br>The entertainment tabloid said that it would break the news of gossip in the near future.
	a.	明星听说了粉丝们*好奇*狗仔队获得了**哪些内幕**。<br>The star heard the fans *were curious about* **which inside dope** the paparazzi had gotten.
	b.	明星听说了粉丝们*认为*狗仔队获得了**哪些内幕**。<br>The star heard **which inside dope** the fans *thought* the paparazzi had gotten.
	c.	明星听说了粉丝们*料到*狗仔队获得了**哪些内幕**。<br>The star heard the fans *foresaw* **which inside dope** the paparazzi had gotten. (low)<br>The star heard **which inside dope** the fans *expected* the paparazzi had gotten. (high)
	d.	明星听说了粉丝们*帮助*狗仔队获得了**哪些内幕**。<br>The star heard **which inside dope** the fans had *helped* the paparazzi to get.
	Question	这则新闻涉及了许多国家机密。(错)<br>This news involves large amounts of national secrets. (False)

8.	Context	教育部在进行小学教育改革，<br>The country is having a reform on primary education.
	a.	家长知道老师们*在打听*教育部增加了**哪些科目**。<br>The parents knew the teachers *were asking* **which subjects** the Ministry of Education had added.
	b.	家长知道老师们*确信*教育部增加了**哪些科目**。<br>The parents knew **which subjects** the teachers *believed* the Ministry of Education had added.
	c.	家长知道老师们*查出了*教育部增加了**哪些科目**。<br>The parents knew the teachers *found out* **which subjects** the Ministry of Education had added. (low)<br>The parents knew **which subjects** the teachers *found out* the Ministry of Education had added. (high)
	d.	家长知道老师们*支持*教育部增加了**哪些科目**。<br>The parents knew **which subjects** the teachers had *supported* the Ministry of Education to add.
	Question	教育部对课程进行了一些改革。(对)<br>The ministry of education did some reform on the curriculum. (True)

9.	Context.	在一档综艺节目录制现场，

		In a variety show recording scene,
	a.	观众听到主持人*询问*嘉宾们提出了**哪些观点**。<br>the audience heard the host *enquired about* **which points** the guest had made.
	b.	观众听到主持人*断言*嘉宾们提出了**哪些观点**。<br>the audience heard **which points** the host *asserted* the guest had made.
	c.	观众听到主持人*忘记了*嘉宾们提出了**哪些观点**。<br>the audience heard the host *forgot* **which points** the guest had made. (low)<br>the audience heard **which points** the host *forgot* the guest had made. (high)
	d.	观众听到主持人*怂恿*嘉宾们提出了**哪些观点**。<br>the audience heard **which points** the host had *incited* the guest to make.
	Question.	这档节目没有主持人和观众。(错)<br>This show has no host or audience. (False)

10.	Context	在小区业主的强烈要求下,<br>Under the strong request of the property owners,
	a.	物业透露了开发商*在调查*住户们放弃了**哪些权利**。<br>the property management company revealed the developers *were investigating* **which rights** the residents had given up.
	b.	物业透露了开发商*断定*住户们放弃了**哪些权利**。<br>the property management company revealed **which rights** the developers *concluded* the residents had given up.
	c.	物业透露了开发商*查明了*住户们放弃了**哪些权利**。<br>the property management company revealed the developers *found out* **which rights** the residents had given up. (low)<br>the property management company revealed **which rights** the developers *found out* the residents had given up. (high)
	d.	物业透露了开发商*欺骗*住户们放弃了**哪些权利**。<br>the property management company revealed **which rights** the developers had *deceived* the residents to give up.
	Question	物业拒绝向业主提供消息。(错)<br>The property management company refused to reveal anything to the property owners. (False)

11.	Context	一起受贿案件将重新审理,<br>A case of bribery will be reopened.
	a.	律师猜到了检察官*想弄清*贪污犯隐藏了**哪些证据**。<br>The lawyer guessed out the prosecutor *wanted to find out* **which evidence** the embezzler had hidden.
	b.	律师猜到了检察官*认定*贪污犯隐藏了**哪些证据**。<br>The lawyer guessed out **which evidence** the prosecutor *affirmed* the embezzler had hidden.
	c.	律师猜到了检察官*揭露了*贪污犯隐藏了**哪些证据**。<br>The lawyer guessed out the prosecutor *revealed* **which evidence** the embezzler had hidden. (low)<br>The lawyer guessed out **which evidence** the prosecutor *revealed* the embezzler had hidden. (high)
	d.	律师猜到了检察官*帮助*贪污犯隐藏了**哪些证据**。<br>The lawyer guessed out **which evidence** the prosecutor *had helped*

		the embezzler to hide.
	Question	这起案件被法院认定是凶杀案。(错)<br>This case was deemed a homicide by the court. (False)

12.	Context	在对各村状况的进行总结时,<br>When reviewing the situation of each village,
	a.	县长记得村支书*在调查*村民们种植了**哪些作物**。<br>the county magistrate remembered the village leader *was investigating* **which crops** the villagers had planted.
	b.	县长记得村支书*号称*村民们种植了**哪些作物**。<br>the county magistrate remembered **which crops** the village leader *claimed* the villagers had planted.
	c.	县长记得村支书*汇报了*村民们种植了**哪些作物**。<br>the county magistrate remembered the village leader *reported* **which crops** the villagers had planted. (low)<br>the county magistrate remembered **which crops** the village leader *reported* the villagers had planted. (high)
	d.	县长记得村支书*鼓励*村民们种植了**哪些作物**。<br>the county magistrate remembered **which crops** the village leader had *encouraged* the villagers to plant.
	Question	县长对各村情况有一定了解。(对)<br>The county magistrate had a certain grasp of each village's situation. (True)

13.	Context	在一起文物造假案中,<br>In a case of counterfeiting cultural relics,
	a.	媒体公布了收藏家*在研究*拍卖行伪造了**哪些证书**。<br>The media revealed the collectors *were researching* **which certificates** the auction house had forged.
	b.	媒体公布了收藏家*断定*拍卖行伪造了**哪些证书**。<br>The media revealed **which certificates** the collectors *concluded* the auction house had forged.
	c.	媒体公布了收藏家*发现了*拍卖行伪造了**哪些证书**。<br>The media revealed the collectors *discovered* **which certificates** the auction house had forged. (low)<br>The media revealed **which certificates** the collectors *discovered* the auction house had forged. (high)
	d.	媒体公布了收藏家*指使*拍卖行伪造了**哪些证书**。<br>The media revealed **which certificates** the collectors had *instigated* the auction house to forge.
	Question	这起造假案没有得到媒体关注。(错)<br>This forgery received little media attention. (False)

14.	Context	在各部门提交的备忘录中,<br>In the memorandum submitted by the various departments,
	a.	主编看到了小组长*打探*实习生完成了**哪些任务**。<br>the editor-in-chief saw the team leader *inquired about* **which tasks** the interns had completed.
	b.	主编看到了小组长*认为*实习生完成了**哪些任务**。<br>the editor-in-chief saw **which tasks** the team leader *deemed* the interns had completed.
	c.	主编看到了小组长*报告了*实习生完成了**哪些任务**。<br>the editor-in-chief saw the team leader *reported* **which tasks** the

		interns had completed. (low)<br>the editor-in-chief saw **which tasks** the team leader *reported* the interns had completed. (high)
	d.	主编看到了小组长*指导*实习生完成了**哪些任务**。<br>the editor-in-chief saw **which tasks** the team leader had *guided* the interns to complete.
	Question	部门中有一些实习生。(对)<br>There are some interns in the departments. (True)
15.	Context	在召开工作会议时大家发现,<br>During the work meeting, people realized
	a.	主播不知道赞助商*好奇*电视台取消了**哪些节目**。<br>the anchorman did not know the sponsor *was curious about* **which shows** the station had canceled.
	b.	主播不知道赞助商*以为*电视台取消了**哪些节目**。<br>the anchorman did not know **which shows** the sponsor *thought* the station had canceled.
	c.	主播不知道赞助商*公布了*电视台取消了**哪些节目**。<br>the anchorman did not know the sponsor *announced* **which shows** the station had canceled. (low)<br>the anchorman did not know **which shows** the sponsor *announced* the station had canceled. (high)
	d.	主播不知道赞助商*要求*电视台取消了**哪些节目**。<br>the anchorman did not know **which shows** the sponsor had *required* the station to cancel.
	Question	这家电视台有赞助商赞助。(对)<br>This TV station has a sponsor. (True)
16.	Context	在商业机密泄露案的调查中,<br>In the investigation of the leakage of commercial secrets,
	a.	警察知道董事长*想了解*清洁工偷走了**哪些文件**。<br>the police knew the chairman *wanted to know* **which documents** the janitor had stolen.
	b.	警察知道董事长*坚称*清洁工偷走了**哪些文件**。<br>the police knew **which documents** the chairman *asserted* the janitor had stolen.
	c.	警察知道董事长*发现了*清洁工偷走了**哪些文件**。<br>the police knew the chairman *discovered* **which documents** the janitor had stolen. (low)<br>the police knew **which documents** the chairman *discovered* the janitor had stolen. (high)
	d.	警察知道董事长*指使*清洁工偷走了**哪些文件**。<br>the police knew **which documents** the chairman had *instigated* the janitor to stole.
	Question	警方已经介入了这起商业案件。(对)<br>Police have been involved in this commercial case. (True)
