# Supplementary material for: Processing covert dependency: An eye-tracking study of scope interpretations of embedded Wh-questions in Mandarin
Source: PLoS One. 2023 May 18;18(5):e0285873. doi: 10.1371/journal.pone.0285873 (PMC10194922; doi:10.1371/journal.pone.0285873)
Supplement: S1 File — (ZIP) [file pone.0285873.s001.zip › supporting infomration/Norming Results.pdf]

Norming Results

**The *好奇 haoqi* （'wonder'） type**			
**Interrogative complement**		**Declarative complement**	
**Sentence**	**Rating**	**Sentence**	**Rating**
经纪人*想知道*狗仔队偷拍了**哪些照片**。<br>The agent *wanted to know* which photos the paparazzi had taken.	4.71	*经纪人*想知道*狗仔队偷拍了**一些照片**。<br>*The agent *wanted to know* the paparazzi had taken some photos.	1.62
市领导*弄不清*博物馆收藏了**哪些古董**。<br>The municipal leaders *were confused about* **which antiques** the museum had collected.	4.62	*市领导*弄不清*博物馆收藏了**那些古董**。<br>*The municipal leaders *were confused about* the museum had collected **those antiques**.	1.86
群众们*想了解*市政府禁止了**哪些行为**。<br>The people *wanted to know* **which behaviors** the city government had prohibited.	4.71	*群众们*想了解*市政府禁止了**这些行为**。<br>*The people *wanted to know* the city government had prohibited **which behaviors**.	1.81
赞助商*好奇*电视台取消了**哪些节目**。<br>The sponsors *were curious about* **which shows** the stations had canceled.	4.67	*赞助商*好奇*电视台取消了**那些节目**。<br>*The sponsors *were curious about* the stations had canceled **those shows**.	2.19
古人类*在探索*外星人建造了**哪些城市**。<br>The ancient humans *were exploring* **which cities** the aliens had built.	4.10	*古人类*在探索*外星人建造了**那些城市**。<br>*The ancient humans *were exploring* the aliens had built **those cities**.	1.71
老师们*在打听*教育部增加了**哪些科目**。<br>The teachers *were asking* **which subjects** the Ministry of Education had added.	4.71	*老师们*在打听*教育部增加了**这些科目**。<br>*The teachers *were asking* the Ministry of Education had added **these subjects**.	2.05
主持人*询问*嘉宾们提出了**哪些观点**。<br>The host *enquired about* **which points** the guest had made.	4.29	*主持人*询问*嘉宾们提出了**那些观点**。<br>*The host *enquired about* the guest had made **those points**.	2.10
警察们*在调查*住户们丢失了**哪些财物**。<br>The police *were investigating* **which properties** the residents had lost.	4.48	*警察们*在调查*住户们丢失了**一些财物**。<br>*The police *were investigating* the residents had lost **some properties**.	2.14
检察官*想弄清*贪污犯隐藏了**哪些证据**。<br>The prosecutor *wanted to find out* **which evidence** the embezzler had hidden.	4.67	*检察官*想弄清*贪污犯隐藏了**那些证据**。<br>*The prosecutor *wanted to find out* the embezzler had hidden **this evidence**.	2.10
收藏家*在研究*拍卖行伪造了**哪些证书**。<br>The collectors *were researching* **which certificates** the auction house had forged.	3.95	*收藏家*在研究*拍卖行伪造了**这些证书**。<br>The collectors *were researching* the auction house had forged **these certificates**.	2.24
小组长*打探*实习生完成了哪些任务。<br>The team leader *inquired about* **which tasks** the interns had completed.	4.62	*小组长*打探*实习生完成了**那些任务**。<br>*The team leader *inquired about* the interns had completed **those tasks**.	1.90

**The *相信 xiangxin*（'believe'） type**			
**Interrogative complement**		**Declarative complement**	
**Sentence**	**Rating**	**Sentence**	**Rating**
*研究员*怀疑*环保队采集了**哪些树种**。	2.00	研究员*怀疑*环保队采集了**一些树种**。	4.43

*The researchers *suspected* **which tree species** the environmental protection team had collected.		The researchers *suspected* the environmental protection team had collected **some tree species**.	
*市领导*以为*博物馆收藏了**哪些古董**。<br>*The municipal leaders *believed* **which antiques** the museum had collected.	1.86	市领导*以为*博物馆收藏了**那些古董**。<br>The municipal leaders *believed* the museum had collected **those antiques**.	4.43
*群众们*相信*市政府禁止了**哪些行为**。<br>*The people *believed* **which behaviors** the city government had prohibited**.**	2.14	群众们*相信*市政府禁止了**那些行为**。<br>The people *believed* the city government had prohibited **these behaviors**.	4.33
*家长们*觉得*学生们学习了**哪些知识**。<br>*The parents *thought* **which knowledge** the students had learned.	1.76	家长们*觉得*学生们学习了**这些知识**。<br>The parents *thought* the students had learned **this knowledge**.	4.48
*总经理*认为*程序员删除了**哪些资料**。<br>*The general manager *believed* **which data** the programmers had deleted.	2.00	总经理*认为*程序员删除了**那些资料**。<br>The general manager *believed* the programmers had deleted **those data**.	4.38
*古人类*坚信*外星人建造了**哪些城市**。<br>*The ancient humans *believed* **which cities** the aliens had built.	2.05	古人类*坚信*外星人建造了**这些城市**。<br>The ancient humans *believed* the aliens had built **these cities**.	4.43
*老师们*确信*教育部增加了**哪些科目**。<br>*The teachers *believed* **which subjects** the Ministry of Education had added.	2.62	老师们*确信*教育部增加了**那些科目**。<br>The teachers *believed* the Ministry of Education had added **those subjects**.	4.43
*主持人*断言*嘉宾们提出了**哪些观点**。<br>*The host *asserted* **which points** the guest had made.	2.29	主持人*断言*嘉宾们提出了**那些观点**。<br>The host *asserted* the guest had made **those points**.	3.71
*收藏家*断定*拍卖行伪造了**哪些证书**。<br>*The collector *concluded* **which certificates** the auction house had forged.	2.24	收藏家*断定*拍卖行伪造了**一些证书**。<br>The collector *concluded* the auction house had forged **some certificates**.	4.67
*检察官*认定*贪污犯隐藏了**哪些证据**。<br>*The prosecutor *affirmed* **which evidence** the embezzler had hidden.	2.72	检察官*认定*贪污犯隐藏了**那些证据**。<br>The prosecutor *affirmed* the embezzler had hidden **this evidence**.	4.53
*村支书*号称*村民们种植了**哪些作物**。<br>*The village leader *claimed* **which crops** the villagers had planted.	2.00	村支书*号称*村民们种植了**这些作物**。<br>The village leader *claimed* the villagers had planted **these crops**.	4.19

The *知道 zhidao* ('know') type

**Interrogative complement Sentence**	**Rating**	**Declarative complement Sentence**	**Rating**
研究员*宣布了*环保队采集了**哪些树种**。<br>The researchers *announced* **which tree species** the environmental protection team had collected.	3.71	研究员*宣布了*环保队采集了**一些树种**。<br>The researchers *announced* the environmental protection team had collected **some tree species**.	3.14
市领导*隐瞒了*博物馆收藏了**哪些古董**。<br>The municipal leaders *concealed* **which antiques** the museum had collected.	3.14	市领导*隐瞒*博物馆收藏了**那些古董**。<br>The municipal leaders *concealed* the museum had collected **those antiques**.	3.57
观众*听到*嘉宾们提出了**哪些观点**。<br>The people *heard* **which behaviors** the city government had prohibited.	3.85	观众*听到*嘉宾们提出了**那些观点**。<br>The people *heard* the city government had prohibited **those behaviors**.	4.48

家长们*清楚*学生们学习了**哪些知识**。<br>The parents *understood* **which knowledge** the students had learned.	4.62	家长们*清楚*学生们学习了**这些知识**。<br>The parents *understood* the students had learned **this knowledge**.	4.43
警察*知道*清洁工偷走了**哪些文件**。<br>The police *knew* **which documents** the janitor had stolen.	4.52	警察*知道*清洁工偷走了**那些文件**。<br>The police *knew* the janitor had stolen **those documents**.	4.57
古人类*明白*外星人建造了**哪些城市**。<br>The ancient humans *knew* **which cities** the aliens had built.	3.71	古人类*明白*外星人建造了**那些城市**。<br>The ancient humans *knew* the aliens had built **those cities**.	3.90
粉丝们*料到了*狗仔队获得了**哪些内幕**。<br>The fans *foresaw* **which inside dope** the paparazzi had gotten.	3.78	粉丝们*料到了*狗仔队获得了**一些内幕**。<br>The fans *foresaw* the paparazzi had gotten **some inside dope.**	3.94
老师们*查出了*教育部增加了**哪些科目**。<br>The teachers *found out* **which subjects** the Ministry of Education had added.	4.19	老师们*查出*教育部增加了**一些科目**。<br>The teachers *found out* the Ministry of Education had added **some subjects**.	4.43
主持人*忘记了*嘉宾们提出了**哪些观点**。<br>The host *forgot* **which points** the guest had made.	4.43	主持人*忘记了*嘉宾们提出了**这些观点**。<br>The host *forgot* the guest had made **those points**.	3.29
警察们*查明了*住户们丢失了**哪些财物**。<br>The police *found out* **which properties** the residents had lost.	4.38	警察们*查明了*住户们丢失了**一些财物**。<br>The police *found out* the residents had lost **some properties**.	3.81
检察官*揭露了*贪污犯隐藏了**哪些证据**。<br>The prosecutor *revealed* **which evidence** the embezzler had hidden.	3.76	检察官*揭露了*贪污犯隐藏了**一些证据**。<br>The prosecutor *revealed* the embezzler had hidden **some evidence**.	3.10
村支书*汇报了*农民们种植了**哪些作物**。<br>The village leader *reported* **which crops** the villagers had planted.	4.10	村支书*汇报*农民们种植了**这些作物**。<br>The village leader *reported* the villagers had planted **these crops**.	3.14
收藏家*发现了*拍卖行伪造了**哪些证书**。<br>The collectors *discovered* **which certificates** the auction house had forged.	3.67	收藏家*发现*拍卖行伪造了**一些证书**。<br>The collectors *discovered* the auction house had forged **some certificates**.	4.76
小组长*报告了*实习生完成了**哪些任务**。<br>The team leader *reported* **which tasks** the interns had completed.	4.24	小组长*报告了*实习生完成了**这些任务**。<br>The team leader *reported* the interns had completed **these tasks**.	3.67
媒体*公布了*收藏家伪造了**哪些证书**。<br>The press *announced* **which certificates** the collector had forged.	4.05	媒体*公布了*收藏家伪造了**这些证书**。<br>The press *announced* the collector had forged **these certificates**.	3.67
记者*报道了*环保队采集了**哪些树种**。<br>The reporter *reported* **which tree species** the environmental protection team had collected.	4.57	记者*报道了*环保队采集了**一些树种**。<br>The reporter *reported* the environmental protection team had collected **some tree species**.	4.29
市长*总结了*市政府禁止了**哪些行为**。<br>The mayor *summarized* **which behaviors** the city government had prohibited.	3.95	市长*总结了*市政府禁止了**一些行为**。<br>The mayor *summarized* the city government had prohibited **some behaviors**.	na
秘书*猜出了*程序员删除了**哪些资料**。<br>The secretary *guessed out* **which data** the programmers had deleted.	4.38	秘书*猜出*程序员删除了**一些资料**。<br>The secretary *guessed out* the programmers had deleted **some data**.	4.71
明星*听说了*粉丝们偷拍了**哪些照片**。	3.05	明星*听说了*粉丝们偷拍了**一些照片**。	3.94

The star *heard* **which photos** the fans had gotten.		The star *heard* the fans had gotten **some photos**.	
物业*透露了*住户们丢失了**哪些财物**。<br>The property management company revealed **which properties** the residents had lost.	4.24	物业*透露了*住户们丢失了**一些财物**。<br>The property management company revealed the residents had lost **some properties**.	3.57
律师*猜到了*贪污犯隐藏了**哪些证据**。<br>The lawyer *guessed out* **which evidence** the embezzler had hidden.	4.19	律师*猜到*贪污犯隐藏了**那些证据**。<br>The lawyer *guessed out* the embezzler had hidden **that evidence**.	4.05
县长*记得*村民们种植了**哪些作物**。<br>The county magistrate *remembered* **which crops** the villagers had planted.	4.57	县长*记得*村民们种植了**那些作物**。<br>The county magistrate *remembered* the villagers had planted **those crops**.	3.86
主编*看到了*实习生完成了**哪些任务**。<br>The editor-in-chief *saw* **which tasks** the interns had completed.	4.10	主编*看到*实习生完成了**那些任务**。<br>The editor-in-chief *saw* the interns had completed **those tasks**.	4.52
主播*不知道*电视台取消了**哪些节目**。<br>The anchorman did not know **which shows** the station had canceled.	4.62	主播*不知道*电视台取消了**那些节目**。<br>The anchorman did not know the station had canceled **those shows**.	3.90

The 帮助 *bangzhu* （'help'） type

Interrogative complement Sentence	Rating	Interrogative complement Sentence	Rating
*研究员*协助*环保队采集了**哪些树种**。<br>The researchers *helped* the conservation team to collect **which tree species**.	1.90	研究员*协助*环保队采集了**那些树种**。<br>The researchers *helped* the conservation team to collect **those tree species**.	4.57
*市领导*资助*博物馆收藏了**哪些古董**。<br>*The municipal leaders *subsidized* the museum to collect **which antiques**.	1.95	市领导*资助*博物馆收藏了**这些古董**。<br>The municipal leaders *subsidized* the museum to collect **those antiques**.	4.71
*群众们*说服*市政府禁止了**哪些行为**。<br>*The people *persuaded* the city government to prohibit **which behaviors**.	1.90	群众们说服市政府禁止了**那些行为**。<br>The people *persuaded* the city government to prohibit **those behaviors**.	4.67
*检察官*帮助*贪污犯隐藏了**哪些证据**。<br>*The prosecutor *helped* the embezzler to hide **which evidence**.	2.71	检察官*帮助*贪污犯隐藏了**这些证据**。<br>The prosecutor *helped* the embezzler to hide **this evidence**.	4.76
*总经理*串通*程序员删除了**哪些资料**。<br>*The general manager *colluded with* the programmers to delete **which data**.	2.00	总经理*串通*程序员删除了**一些资料**。<br>The general manager *colluded with* the programmers to delete **some data**.	4.62
*古人类*配合*外星人建造了**哪些城市**。<br>*The ancient humans *cooperated with* the aliens to build **which cities**.	2.24	古人类*配合*外星人建造了**这些城市**。<br>The ancient humans *cooperated with* the aliens to build **these cities**.	4.81
*老师们*支持*教育部增加了**哪些科目**。<br>*The teachers *supported* the Ministry of Education to add **which subjects**.	1.86	老师们*支持*教育部增加了**一些科目**。<br>The teachers *supported* the Ministry of Education to add **some subjects**.	3.57
*主持人*怂恿*嘉宾们提出了**哪些观点**。<br>*The host *incited* the guest to make **which points**.	2.19	主持人*怂恿*嘉宾们提出了**那些观点**。<br>The host *incited* the guest to make **those points**.	4.43
*收藏家*指使*拍卖行伪造了**哪些证书**。	2.43	收藏家*指使*拍卖行伪造了**一些证书**。	4.67

*The collectors *instigated* the auction house to forge **which certificates**.		The collectors *instigated* the auction house to forge **those certificates**.	
*小组长*指导*实习生完成了**哪些任务**。<br>*The team leader *guided* the interns to complete **which tasks**.	2.43	小组长*指导*实习生完成了**这些任务**。<br>The team leader *guided* the interns to complete **these tasks**.	4.67
*赞助商*要求*电视台取消了**哪些节目**。<br>*The sponsors *required* the stations to cancel **which shows**.	2.05	赞助商*要求*电视台取消了**那些节目**。<br>The sponsors *required* the stations to cancel **those shows**.	4.62
*村支书*鼓励*村民们种植了**哪些作物**。<br>*The village leader *encouraged* the villagers to plant **which crops**.	2.24	村支书*鼓励*村民们种植了**这些作物**。<br>The village leader *encouraged* the villagers to plant **these crops**.	4.48
